# Function and Evolution of Nuclear Receptors in Environmental-Dependent Postembryonic Development

**DOI:** 10.3389/fcell.2021.653792

**Published:** 2021-06-10

**Authors:** Jan Taubenheim, Constantin Kortmann, Sebastian Fraune

**Affiliations:** Zoology and Organismic Interactions, Heinrich Heine University Düsseldorf, Düsseldorf, Germany

**Keywords:** nuclear receptor (NR), thyroid hormone, ecdysone, estrogen, metamorphosis, sexual maturation

## Abstract

Nuclear receptors (NRs) fulfill key roles in the coordination of postembryonal developmental transitions in animal species. They control the metamorphosis and sexual maturation in virtually all animals and by that the two main environmental-dependent developmental decision points. Sexual maturation and metamorphosis are controlled by steroid receptors and thyroid receptors, respectively in vertebrates, while both processes are orchestrated by the ecdysone receptor (EcR) in insects. The regulation of these processes depends on environmental factors like nutrition, temperature, or photoperiods and by that NRs form evolutionary conserved mediators of phenotypic plasticity. While the mechanism of action for metamorphosis and sexual maturation are well studied in model organisms, the evolution of these systems is not entirely understood and requires further investigation. We here review the current knowledge of NR involvement in metamorphosis and sexual maturation across the animal tree of life with special attention to environmental integration and evolution of the signaling mechanism. Furthermore, we compare commonalities and differences of the different signaling systems. Finally, we identify key gaps in our knowledge of NR evolution, which, if sufficiently investigated, would lead to an importantly improved understanding of the evolution of complex signaling systems, the evolution of life history decision points, and, ultimately, speciation events in the metazoan kingdom.

## The Nuclear Receptor Family

Metazoans depend, unlike unicellular organisms, on regulative mechanisms to coordinate different tissues and cells. Nuclear receptors (NRs) mediate this coordination and provide a direct link between extracellular signaling molecules and the transcriptional response by recognizing special DNA sequences, the hormone response elements (HREs). NRs form a family of metazoan proteins that regulate fundamental biological processes like cell proliferation, development, metabolism, and reproduction (Laudet, 1997; Fahrbach et al., 2012; Sever and Glass, 2013), while integrating environmental inputs, which renders them key molecules for phenotypic plasticity (Gilbert and Epel, 2015).

Nuclear receptors respond to small, mostly hydrophobic molecules. These include hormones, produced in special tissues, endogenous or exogenous metabolites, or xenobiotics, which are detrimental to the organism (Escriva et al., 2004). Ligands for the receptors can thereby enter the cell either by diffusion due to their hydrophobic nature or by active transport *via* specific transporter (Schweizer et al., 2014; Okamoto et al., 2018). After entering the cell, the ligands are recognized by their NR, which are able to mediate transcriptional regulation upon binding (Sever and Glass, 2013). However, the ligands’ action might be complemented by recognition of membrane receptors, which are often associated with non-transcriptomic regulations (Filardo and Thomas, 2012). As soon as the NRs are activated by their ligand, they regulate the transcription of target genes.

Most importantly, NRs are involved in virtually all major postembryonal developmental steps in metazoans. We will here review the current knowledge of NR involvement in major life history changes, mainly morphogenesis, in multicellular animals and try to draw conclusions on the evolution of these developmental steps. Furthermore, we will stress the importance of NRs for phenotypic plasticity by the integration of environmental signals into the developmental pathways lying beneath these postembryonal morphological adaptations.

### Structure of Nuclear Receptors

**FIGURE 1:**
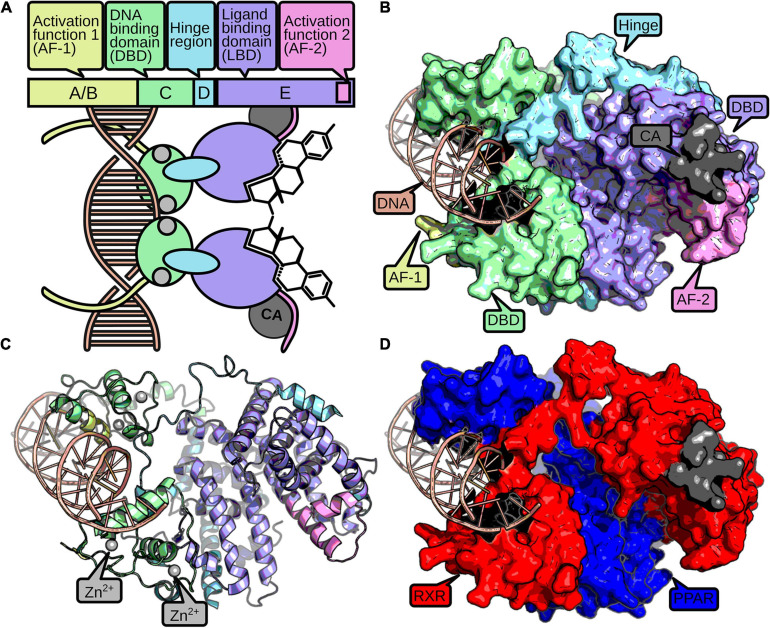
The structure of a nuclear receptor is defined by up to five domains. The AF-1 domain (yellow) can mediate ligand-independent transcriptional regulation. The DBD consists of two zinc-finger domains (green), which are stabilized by zinc ions (gray). The zinc-finger domains recognize and bind to specific DNA-binding sites (red). A hinge region (blue) connects the DBD and the LBD (purple). The LBD usually binds a small hydrophobic ligand, which induces dimerization and a conformational change in the AF-2 domain (pink). Active AF-2 stabilizes the binding to the DNA by recruiting coactivators (CA, dark gray) and mediates transcriptional activity. A schematic representation of a NR homodimer is displayed in **(A)**, while a surface and cartoon representation of an RXR-PPAR heterodimer crystallization (PDB: 3DZY) (Chandra et al., 2008) is given in **(B,C)**, respectively, following the same color scheme as above. For better discrimination of the two dimers, **(D)** displays PPAR in blue and RXR in red.

Nuclear receptors consist of up to four domains that fulfill different modular functions (Figure 1). The C-domain, also referred to as DNA-binding domain (DBD) is stabilized by two zinc fingers, necessary for identification and binding to specific response elements in the DNA (Kumar and Thompson, 1999). The E-domain includes the ligand-binding domain (LBD) and enables the NRs to regulate the transcription after ligand binding. The C-terminal part of the LBD contains an activation function 2 (AF-2) subdomain and enhances the ligand-dependent transcription by binding to coactivation factors (Wärnmark et al., 2003). The A/B-domain, or N-terminal domain, is variable and comprises an activation function-1 motif (AF-1) in most NR proteins that may induce a ligand-independent transcription. The diverse D-domain is often referred to as “hinge” due to its function as a connector between DBD and LBD (Fahrbach et al., 2012).

Nuclear receptors developed diverse structural mechanisms to stabilize the active conformation together with the ligand (Germain et al., 2006). Usually, a heat shock protein dissociates from the receptor upon ligand binding, which enables homo- or heterodimerization with other NRs and is accompanied with translocation into the nucleus for cytosolic NRs. Furthermore, ligands change the conformation of the AF-2 domain by binding to the NR’s allosteric center, which supports the binding of NRs with additional coactivators and inhibits association of corepressors (Sever and Glass, 2013). Hence, the possibility to form the active conformation is important to activate expression of target genes and therefore for the NR’s function (Kumar and Thompson, 1999).

## Evolution of NRs

**FIGURE 2:**
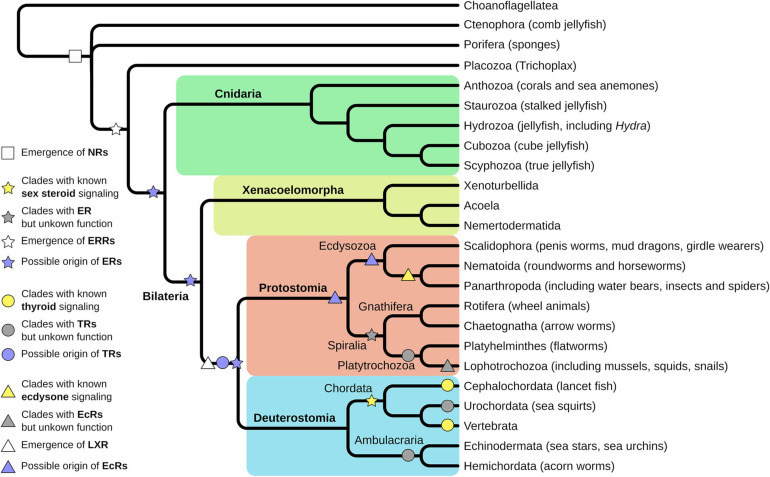
Evolution of nuclear receptors (NRs) starts at the base of metazoan life. Porifera are the first animal clade where functional NRs emerged (white square). *Trichoplax* evolved an estrogen-related receptor (ERR), an orphan receptor, and close homolog to the estrogen receptor (ER) (white star). Evolution of a ligand-binding ER is proposed before the split of Protostomia and Deuterostomia, thus is located either in the Cnidarian or the Xenacoelomorpha lineage (purple star). Experimental support for ligand-binding ERs has been found in Annelida and Rotifers, thus in the Sprialia lineage within the Protostomia (gray star). Fully described and functional estrogen signaling exists in Vertebrata and Cephalochordata, thus in the Chordata lineage (yellow star). Homologs for thyroid receptors (TRs) have been identified in Lophotrochozoa, Urochordata, and Ambulacraria (gray circles) but were functionally characterized only in Cephalochordata and Vertebra (yellow circles). However, emergence of the TR was proposed at the base of Protostomia and Deuterostomia (purple circle). Ecdysone receptor (EcR) signaling has been functionally described in Panarthropoda and to some extent in Nematoida (yellow triangle) but homologs have been identified in different Lophotrochozoa (gray triangle). Hence, the emergence of EcR can be presumed at the base of Protostomia, but at least in the Ecdysozoan clade (purple triangles). The closely related LXR receptor is assumed to have emerged at the base of Protostomia and Deuterostomia (white triangle).

Understanding the evolution of NRs will help to decipher the evolution of different life history (e.g., larval-adult stage vs. direct development) as the developmental processes are regulated by members of the NR family. Thus, it also has direct implication for our understanding of the evolution of new species, because NRs regulate key functions for integration of environmental and endogenous signals into developmental processes and are crucial for correct timing of developmental transitions. Looking at the diversity of animal species, it is striking that seemingly members of all major clades of metazoan life use NRs to regulate these developmental transitions, although different members of the NR family take part. However, the evolutionary origin of NRs lies at the base of metazoan life and is not an inherited feature of earlier single cell evolution (Bridgham et al., 2010; Figure 2).

The sponge *Amphimedon queenslandica* contains only two members of the NR family, both belonging to the NR2 subfamily (the same as RXR, see below) (Bridgham et al., 2010; Figure 2). From here, there exist mainly two different scenarios of NRs’ diversification. A first theory, based on initial phylogenetic analyses, assumed that the ancestral NR functioned as a constitutive transcription factor without binding a ligand. The receptors descendants acquired the capability to bind ligands secondarily and independently, at different times in evolution (Escriva et al., 2000; Fahrbach et al., 2012). This theory is supported by the fact that NRs are binding structurally different ligands in the same subfamily and the orphan receptors (receptors without a known ligand) are widely spread out in the phylogenetic tree. This implies that there is no connection between the evolutionary relationships of NRs and the origin of their ligands. For example, the evolutionary closely related receptors of subfamily I, the thyroid hormone receptors (TRs), the retinoic acid receptors (RARs), the peroxisome proliferator-activated receptors (PPARs), and the vitamin D receptors (VDRs), bind to ligands that derive from entirely different biosynthetic pathways (Escriva et al., 2000). Furthermore, the RARs (NR1) and the retinoid X receptors (RXR; NR2) are evolutionary less related but bind to the same ligand (retinoid acid), which resembles independent convergent evolution (Escriva et al., 2000). This makes sense in the light of evolutionary constraints, which were placed on the LBD of NRs. Many extant NRs function as metabolic sensors, regulating metabolism and thus have to integrate signals, which are specific to the nutrition of the organism (Garcia et al., 2018). This in turn implies that early evolution of NRs was also associated with metabolic regulation. While the metabolic network regulated by the ancient DBD was relatively fixed, nutritional input could change easily during exploration of new ecological niches of the organism. Thus, the DBD was constrained to regulate the metabolic network, while the LBD had to be flexible and maybe was aquired several times independently during evolution of the ligand binding feature of the NRs.

An alternative scenario implies that the ancient NRs may have been lipid sensors, which are receptors with relatively low affinity for a range of hydrophobic molecules like hemes, retinoids, steroids, fatty acids, eicosanoids, and other lipids, that are ingested with nutrition. In fact, the two NRs expressed in the sponge *A. queenslandica* bind long chained fatty acids like palmitic acid (Bridgham et al., 2010). The low affinity binding contrasts them to hormonal receptors that have a high affinity for very specific compounds. During evolution, these multipurpose lipid sensors presumably lost the ligand-based regulation of transcriptional activation secondarily by duplications and neofunctionalization to become what is known as orphan receptors today (Markov and Laudet, 2011). Other receptors specialized to bind particular molecules with a very high affinity and formed hormone specific receptors (Markov and Laudet, 2011). The existence of liganded NRs in early branching phyla underlines this theory and several studies identified different liganded NRs in basal metazoans (Keay and Thornton, 2009; Bridgham et al., 2010; Novotný et al., 2017; Khalturin et al., 2018). Hence, both theories have their reasoning and it seems obvious that LBD and DBD of the receptors show different evolutionary trajectories, given their different subjection to evolutionary constraints. It thus might appear on the molecular level that the two domains evolved as two separate genes. In fact, ancient NRs might have been a product of the fusion of LBD and DBD proteins, as for instance early branching Ctenophora NRs consist only of a LBD but contain no DBD (Reitzel et al., 2011).

Interestingly, although a metazoan innovation, NRs are able to function in nonmetazoan contexts: transfection of NRs into yeast or plants yielded functional receptors, which were able to control transcription (although containing species specific DBDs) (Metzger et al., 1988; Schena and Yamamoto, 1988; Schena et al., 1991). This indicates that NRs evolved bounded to already present regulatory cellular protein interactions, which were adapted to facilitate transcriptional regulation.

In early branching metazoans, at least seven NR subfamilies (NR1–7) with several groups and members exist (Nuclear Receptors Nomenclature Committee, 1999), suggesting a rapid expansion of the family during early metazoan evolution (Bertrand et al., 2004). It is possible to discern two periods of diversification through gene duplication by comparison of different taxa, e.g., arthropods and vertebrates (Escriva et al., 1998; Nuclear Receptors Nomenclature Committee, 1999):

i. The first diversification occurred before the split of Deuterostomia/Protostomia. This led to the appearance of the seven families and their receptors.
ii. The second split created the paralogous groups (e.g., TRα and β, RARα, β, and γ) within the families after bilaterian/pre-bilaterian division, especially in vertebrates.

This pattern is also visible in other gene families like Hox or Ets transcription factors (Escriva et al., 2004). Retinoic acid receptors (RARs and RXRs) regulate the Hox gene transcription in vertebrates, thereby implying a connection between the homeotic genes, that determine the cell identity in the developing embryo, and the NRs, which regulate the cell-to-cell communication (Escriva et al., 1998). Additionally, synteny analysis of the CYP enzymes revealed that the metazoan seeding cluster for the CYP diversity is located close to the Hox gene cluster. CYP enzymes are involved in almost all NR ligand synthesis processes and are virtually always targets of NR regulation, thus form a strong interaction partner in the NR-mediated processes (see below). This might explain the parallel evolution of *hox* genes and CYP enzymes and thus the coevolution of different NRs, Hox, and CYP enzymes (Nelson et al., 2013).

## Steroid Receptors Signaling

### Function of Steroid Receptors

One of the most profound postembryonal developmental transitions in all animals is sexual maturation. Sex determination and maturation are processes, which are highly dependent on sex steroids—androgens, estrogens, and progestogens in vertebrates. These hormones have pleiotropic effects on the individual organism, starting from behavioral changes (Frankl-Vilches and Gahr, 2018), to sexual maturation like gonad development (Hamilton et al., 2017; Fuentes and Silveyra, 2019) and development of secondary sexual traits (Ogino et al., 2018).

The developmental differences upon the stimulation of NRs in the estrogen and ketosteroid receptor subfamily (NR3 subfamily) by steroids are very diverse and species specific, ranging from special appendages in viviparous fish to vocal organ development in amphibians to the development of secondary sexual features in humans (Ogino et al., 2018). To mention all these differences in the sexual development in vertebrate species would go beyond the scope of this review, as sexual development is a highly species specific trait and has been reviewed elsewhere (Valenzuela, 2008; Ogino et al., 2018). However, all these effects are regulated by NRs, which bind a highly specific (nanomolar affinity) steroidal ligand comprising two ER (ERα and ERβ), an androgen (AR), and a progesterone receptor (PR) (Baker, 2019). Of these, the two ERs and the AR play the major roles in postembryonal development by mediation of the development of sex-specific phenotypes and behavior. Steroid signaling is thereby orchestrated by a range of environmental and developmental cues, which again, are highly species specific. Vertebrates include day length and/or body size information to time puberty with season and food availability (Leka-Emiri et al., 2017; Paul et al., 2018; Hanlon et al., 2020), which are the most common sources for environmentally induced variation (phenotypic plasticity).

However, sexual maturation is also associated with the reduction of growth in vertebrates. Usually, the sexual developmental switch is induced after a critical size/weight threshold is reached and the environmental conditions allow for sexual maturation (Hyun, 2013; Leka-Emiri et al., 2017; Hanlon et al., 2020). This in turn causes a cease of growth in the organism and sexual maturation determines the final body size. From an evolutionary perspective, it resembles a switch for resource allocation: from investment in growth to investment in reproduction. This is a delicate switch and highly dependent on the environment as detrimental conditions can cause either increase of developmental speed to reach sexual maturity and ensure offspring before death, or it leads to the deceleration of growth in order to endure unfavorable conditions and postpone development. Larger body sizes are generally associated with higher survival, larger harem sizes, and higher fecundity, but it comes to the expense of higher resource demand, longer developmental times, and more time in potentially vulnerable larval stages (Blanckenhorn, 2000; Kingsolver and Huey, 2008).

### Evolution of Steroid Receptors

The complete NR3 subfamily of NRs consists of estrogen related receptors (ERR, NR3B), 3-ketosteroid receptors (NR3C, containing gluco- and mineralocorticoid, progesterone, and androgen receptors) and estrogen receptors (NR3A). However, the full set of receptors is only present in vertebrates. A genome duplication event in the common ancestors of Gnathostoma (sharks are the first split within the clade) facilitated the diversification of a single steroid receptor (SR) into today’s known receptors for corticosteroids, androgens, progesterones, and aldosterones (Baker, 2019), while an ancestral ER/ERR diversified into the extant ERs and ERRs.

Before further discussion on the topic, we should clarify, that hereafter we use the term ER and ERR within the Protostomia and pre-Bilateria clades, which could be misleading as defining correct orthology is a difficult task. We consider all homologs of the NR3 subfamily as orthologs to either ER or ERR and used the terms depending on the suggested orthology and/or the function of the receptor in the organism. However, correct naming of NR3 receptors in invertebrates is controversial (Markov et al., 2008) and we want explicitly state that orthology to ketosteroid receptors might be just as probable, despite our choice of terminology.

The evolution of sex SRs in invertebrate species is less clear today and especially the origin of estrogen signaling is under debate. Evolutionary earliest evidence for NR3 members can be found in Placozoa where an ERR was identified in *Trichoplax adhaerens* that clusters as an outgroup to vertebrate ERs (Baker, 2008; Novotný et al., 2017; Figure 2). Furthermore, some cnidarian species seem to have retained this receptor, e.g., in *Hydra* (Khalturin et al., 2018), although in others cnidarians like *Nematostella vectensis*, no NR3 subfamily member was identified (Reitzel and Tarrant, 2009). Additionally, an ERR was annotated in the genome of *Hofstenia miamia* (Gehrke et al., 2019), a Xenacoelomorpha [forms a bilaterian sister group to all Deuterostomia and Protostomia (Rouse et al., 2016; Cannon et al., 2016; Figure 2]. The physiological function of these genes and whether they were able to bind a steroid (or other) ligand is unclear to date. However, investigation of the function of these receptors might be rewarding, as knowledge about the metamorphic events in Cnidaria are currently lacking, but seem coordinated by NR signaling (Fuchs et al., 2014).

Within the Protostomia, ERs, and ERRs can be found in Lophotrochozoa (Figure 2). There is evidence for functional sex SRs in three classes of mollusks: Bivalvia, Gastropoda, and Cephalopoda (Köhler et al., 2007). In contrast to vertebrate ERs, they are not activated by estrogen but mediate transcription constitutively. The mollusks ER’s LBD’s allosteric switch became possibly stuck in the agonist position and leads to constitutive transcription (Keay and Thornton, 2009). Although various publications exist where steroids (especially those also active in vertebrates) are reported to influence developmental timing and number of gonads in mollusks and that enzymatic functionality is present (Ketata et al., 2008; Fernandes et al., 2011), there is considerable critique on these reports (Scott, 2012, 2013; Minakata and Tsutsui, 2016; Fodor et al., 2020). It is thus not quite clear, whether Molluska employ steroids to control sexual maturation.

However, ERs that are sensitive to estrogen and endocrine disruptors have been found in annelida, the sister phylum of mollusks (Keay and Thornton, 2009). Keay and Thornton isolated and characterized the NRs from two annelids, *Platynereis dumerilii* and *Capitella capitata*, which are orthologs of mollusk and vertebrate ERs. The annelid ERs show the same functions as vertebrate ERs in estrogen sensitivity and specificity. They recognize classic estrogen responsive elements and activate transcription at low doses of estrogen. Estrogen is produced by the annelids themselves and is therefore not only an environmental factor. The hormones regulate the provisioning of oocytes with vitellogenin during female reproduction and the ERs mediate these effects (Keay and Thornton, 2009). This was a surprising finding, as the estrogen signaling was thought to be a mere vertebrate specific feature.

These results are complemented by studies in rotifers, a sister group to Platytrochozoa (comprising Platyhelminthes and Lophotrochozoa) (Figure 2). In a phylogenetic study, an ER homolog has been identified in different *Brachionus* species (Kim et al., 2017). Furthermore, biochemical studies identified steroid derivatives, a functional progesterone, and an estrogen receptor of the NR3 subfamily in *Brachionus manjavacas* (Stout et al., 2010; Jones et al., 2017). Both seem to be associated with sexual reproduction, leading to the assumption that rotifers employ an estrogen-like signaling pathway to coordinate their reproductive processes.

Within the Platyhelminthes, no NRs of NR3 subfamily could be identified. Thus, this class of proteins seems to have been lost in this phylum (Wu and LoVerde, 2019; Figure 2).

In Ecdysozoa, an ERR has been identified (Bridgham et al., 2010; Fahrbach et al., 2012). It is involved in the downstream regulation of the EcR (see below), but seems not involved in hormonal recognition (Tennessen et al., 2011; Beebe et al., 2020), despite being an integrator of environmental signals (Li et al., 2013) and regulator of metabolism (Beebe et al., 2020).

Taken together, the presence of NR3-family members in Mollusks, Annelida, Rotifers, and Ecdysozoa implies the evolution of the prototype receptor for the vertebrate estrogen in a common ancestor of Protostomia and Deuterostomia. Several questions remain: was the ancestral receptor able to bind a steroid ligand, and are extant receptors in early branching metazoans? And is the extant receptor involved in major developmental processes?

Within the Deuterostomia invertebrates, nearly nothing is known about steroid signaling. There have been some suggestive publications on sex steroid effects in Echinodermata (Köhler et al., 2007), but there is no link to an active NR, or receptor in general (Silvia et al., 2015). The same is true for Tunicata, which seem to regulate sexual maturity mainly through peptidergic signaling (Tello et al., 2005; Matsubara et al., 2019). The best studied NR3 members are those of Cephalochordata. In Branchiostoma, a fully functional SR next to an ER without ligand binding capacity was identified (Callard et al., 2011; Figure 2). It seems likely that all other vertebrate SRs including the functional estrogen receptor diversified from these two genes.

Differences in presence and absence of receptors in different phyla and differences of ligand-binding activity, if a receptor is present, promoted a series of studies, which tried to infer the ancestral state of the receptor and its ligand by using phylogenetic maximum likelihood approaches. This ancestral sequence reconstruction is based on statistical support for most likely amino-acid compositions (or reactions leading to a ligand) of the respective protein/molecule of interest. These can then be cloned and heterologously expressed (or synthesized) to experimentally explore biochemical and signaling properties. For example, an ancestral SR was inferred using inactive lophotrochozoan sequences, but was able to bind steroid derivatives (Thornton, 2003). Furthermore, the ancestral steroid ligand could be reconstructed and is able to bind and activate an ancestral receptor (Markov et al., 2017), although in micromolar range, which is weaker compared to hormones. Additionally, these studies contributed to our understanding how transcription factor binding specificity to its recognition DNA sequence (McKeown et al., 2014) and ligand-receptor specificity evolved (Eick et al., 2012). Both need predominantly mutations to inhibit specific binding in order to escape an evolutionary trap, which is formed by the already present function of the protein. Otherwise, newly gained functions would readily interfere with (vital) present functions of the receptor. In case of the ligand recognition function of the receptor: specificity is achieved by excluding ligands with missing features, rather than recognizing all features of a given ligand for NRs (Eick et al., 2012). This might also explain the broad ranges of xenobiotics recognized by different NRs and the final evolution of highly sensitive hormone receptors (which acquired more feature recognition sites). Finally, these results all point to the evolution of steroid binding SR, which were derived from less-specific ligand binding NRs, which diversified and specified during evolution in the different clades.

However, although our knowledge is quite deep in certain details of SR evolution, experimental evidence of the function is missing in many phyla. The Lophotrochozoan clade in particular seems to hold much information about the evolution of ERs as functional as well as nonfunctional ERs exist. Similar is true for the evolution of the promiscuous NR, which forms the ancestor to all other ERs. Neither a definitive protein, ligand, nor function have been identified or studied in extant organisms in one of the sister groups to the Protostomia-Deuterostomia. Exploration of these animal clades, however, contains important functional information about regulation and consequences of activation of this receptor, which is needed to understand the evolutionary constraints of the molecular changes in the ERs.

## Thyroid Receptor Signaling

### Function of Thyroid Receptors

Thyroid hormones are the major players for induction of metamorphosis in vertebrates (Laudet, 2011) and control many metabolic functions in human (Mullur et al., 2014). The function of this signaling cascade is well understood in vertebrate systems, where basically all poikilotherm species undergo a metamorphic event in their life history controlled by TH signaling. This event is often associated with dramatic morphological and physiological changes, for example, the transition from tadpoles to juvenile frogs, or the transition from benthic blind lamprey larva to pelagic sighted juvenile individuals. It can, however, be more subtle as in fish, where still some debate exists, whether the morphological changes resemble a real metamorphosis (Campinho, 2019).

Amphibian transition from aquatic, mostly herbivore tadpoles to terrestrial, carnivore adults is thereby a textbook example of larva-to-adult metamorphosis. It is one of the best studied postembryonal developmental processes in the vertebrate clade, especially in the clawed frog *Xenopus laevis*. The metamorphosis in *Xenopus* is mediated by TRs (TRα, NR1A1; TRβ, NR1A2) and a peak of the thyroid hormone (TH, here T*3* and T*4*), which coincides with the development of the thyroid gland in the tadpole. The thyroid gland produces the T*4* hormone, which is biologically less active. It first has to be converted to biological active T*3* or is inactivated by Deiodinases (D1–3, D1, and D2 produce T*3*, while D3 deactivates T*4* and T*3*) in a tissue specific manner, resulting in differential response to circulatory TH release in different tissues (Mullur et al., 2014).

**FIGURE 3:**
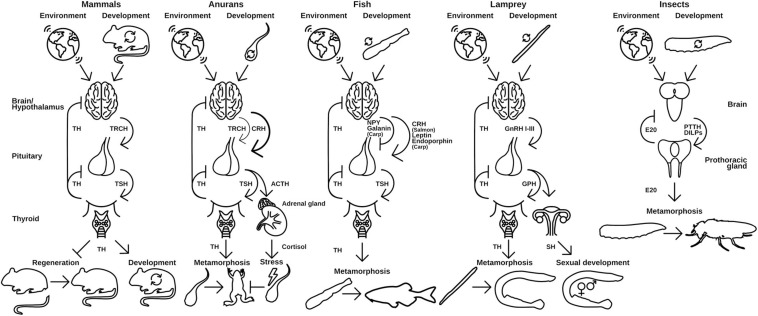
Hypothalamus-pituitary-thyroid axis in different vertebrates use distinct releasing hormones to regulate TH release. Environmental and developmental signals control the release of releasing peptide hormones from the brain, which initiates the release of thyroid-stimulating hormones (TSH) from the pituitary gland. TSH in turn activates the production and release of TH from the thyroid. Released TH initiates and regulates metamorphic events in the animal. A similar regulation is seen in insects and the release of ecdysone (E20). PTTH and DILPS are produced in the brain after developmental and environmental cues, which induce the production of E20 in the PG. TRCH, thyroid releasing hormone; TSH, thyroid-stimulating hormone; CRH, corticotropin-releasing hormone; ACTH, adrenocorticotropic hormone; NPY, neuropeptide Y; GnRH, gonadotropin-releasing hormone; GPH, gonadal pituitary hormones; SH, sex hormones; PTTH, prothoracictropic hormone; DILP, *Drosophila* insulin-like peptides. Parts of the figures were derived from www.phylopic.org and www.svgrepo.com/page/licensing and are public domain or licensed under CC0 1.0.

Tissue specific responses to TH cause a resorption of the tail, growth of the limbs and remodeling of the intestine and nervous system, among other tissue adaptations. Interestingly, TRα and TRβ have contrary functions: while TRα induces growth and cell proliferation in tissues like brain, limb buds, and skin upon TH binding, TRβ causes apoptosis and proteolysis in tail and gills (Mourouzis et al., 2020). However, the metamorphosis of amphibians is not a spontaneous process. It is dependent on the corticotropin releasing hormone (CRH) in the hypothalamus of the tadpole, which induces the release of TSH in the pituitary and consequently induces the TH production in the thyroid gland (Laudet, 2011; Figure 3). This process is dependent on the environmental factors like presence of predators or pond drying and integrates with the general stress response of the animals *via* cortisol (Denver, 2009; Laudet, 2011). In that regard, CRH is not only regulating the TSH release but also the ACTH release in the pituitary, which, as a consequence, additionally regulates cortisol levels in *Xenopus* (Figure 3) leading to de- or acceleration of the development depending on the animal’s developmental stage (Denver, 2009; Laudet, 2011).

Apart from the reoccurring pattern of environmental integration of important developmental steps *via* NR-driven processes, anuran species harbor another interesting feature: nonmetamorphic species. These frogs hatch as small adult variants and skip the tadpole stage. However, it seems that the nonmetamorphic frog *Eleutherodactylus coqui* goes through a morphogenesis-like transition *in ovo*, induced partly by maternal addition of TH to the egg (Laudet, 2011; Laslo et al., 2019). The same seems to be true for different fish and salamander species, which show no obvious metamorphosis (Laudet, 2011). This finding has evolutionary consequences, as it poses the question, whether other nonmetamorphic vertebrates completely lost a larval stage on the ontological level, undergo metamorphosis during embryogenesis by provision of maternal hormones or undergo a cryptic postembryonal metamorphosis. For example, in humans, TH concentrations are correlated with size and growth during embryonic development and are maternally provisioned during the first 4 weeks (Forhead and Fowden, 2014). After that point, it is mostly endogenously produced by the embryo, which resembles the sequence of events in *E. coqui* during embryogenesis. To answer the question regarding the evolution of direct and larval development, it is pivotal to understand the evolution of molecular key regulators in metamorphic events, like NRs.

The molecular implementation of TH activation and release is generally the same for all species: upon environmental and developmental cues, which are received in the hypothalamus, a peptidergic releasing hormone activates the associated pituitary gland (Figure 3). The pituitary releases a thyroid stimulating hormone (TSH) and thus induces TH production in the thyroid gland/endostyle where the signal is received. The TH is released into the circulation and recognized by NRs in target tissues, where it induces tissue specific effects. This axis of regulation is called hypothalamus-pituitary-thyroid axis (HPT-axis) and although the general pattern is the same in all vertebrates, species specific differences in this regulation exist. The metamorphic event is generally associated with a sharp and rather sudden increase in free TH-serum levels leading to induction of larva-to-adult transitions (Laudet, 2011).

The implementation of the releasing hormone in the hypothalamus differs in vertebrate species (Figure 3). While mammals and birds use a specific thyroid releasing hormone (Manzon and Manzon, 2017; Lazcano et al., 2020), amphibians predominantly employ a corticotropin (CRH)-like peptide (Laudet, 2011; Lazcano et al., 2020). For fish, contradicting reports in different species exist, but it seems that the major teleost clades developed specific implementations of the releasing hormone signaling. While Salmonidae seem to also use CRH (Campinho, 2019), Cyprinidae use a combination of leptins, endorphins (both activating), neuropeptide Y, and Galanin (both inhibiting) to control their TH release. Cyclostomes use three different gonadal releasing hormones (GnRHI-III) to regulate the release of TH (Manzon and Manzon, 2017).

Cyclostomata form the sister group to all other vertebrates and are the earliest branching phyla within the Vertebrata (Kuraku and Kuratani, 2006; Miyashita et al., 2019). Extant species of the cyclostomes comprise lampreys and hagfish. Lampreys show metamorphosis from eyeless, filter feeding and benthic living larva to mostly parasitic, pelagic juveniles (Manzon and Manzon, 2017). The transition between larvae and juveniles is controlled by TH signaling, which is in its basics the same as in anurans.

However, a divergence in TH action is present as the TH concentration rises throughout the larval stage followed by a sharp decline that induces the metamorphosis (Leatherland et al., 1990; Youson et al., 1994; Manzon and Manzon, 2017). Hence, the pattern of TH induced metamorphosis has clearly evolved before the emergence of Vertebrata.

In Cyclostomata, the metamorphic event is dependent on two environmental factors: temperature and population densities. While low temperatures inhibit metamorphosis in general, it is the change from cold to warmer temperatures (probably as sensor for seasonal changes) which induces metamorphosis (Leatherland et al., 1990; Holmes and Youson, 1994; Youson et al., 1994). High population densities, however, prevent metamorphosis as it reduces growth of the larvae since a critical size/weight (called conditioning factor in fisheries biology) is needed before metamorphosis is induced (Manzon and Manzon, 2017). Food is generally not a limiting factor and it is unclear why growth is hampered in high populations of lamprey, despite a chemical signal secreted in the water column was suspected (Rodriguez-Munoz et al., 2003). How these environmental cues are integrated in the thyroid signaling pathway is not clear to date, but the molecular signatures associated with this signal integration promises to unravel core mechanisms of developmental plasticity in the vertebrate clade.

In mammals, TH action in development takes place predominantly during embryogenesis, where it promotes growth and maturing of the organs, apart from regulating diverse metabolic functions in the embryo to ensure timely and full development at birth (Forhead and Fowden, 2014; Mourouzis et al., 2020). It has delicate functions in the developing nervous system where minor changes in the concentration of TH during embryogenesis can have impact on the final IQ of humans (Sachs and Buchholz, 2017). Postnatally, THR regulates growth and maturation of muscle and bone, leading to short stature if disturbed after birth. In hyperthyroid conditions, this is caused by rapid skeletal growth and premature fusion of the growth plates in bones, while hypothyroidism causes delayed bone maturation with lower bone mineralization and general skeletal dysplasia (Mourouzis et al., 2020). However, the most pronounced effect of TH in mammalian postnatal development is the loss of regenerative capacity, which coincides with terminal differentiation of myoblasts to myocytes (Lee et al., 2014) and with a peak of TH at birth in human and roughly 7 days after birth in mouse (Wu and LoVerde, 2019). Axon regeneration in the mouse brain is active for the first week after birth, and external addition of TH or removal is reducing and extending this plasticity respectively (Avci et al., 2012). Similarly, heart regeneration is possible in newborn mice but this capability is lost after the TH peak at around 7 days (Hirose et al., 2019). The same authors associated regeneration capacity and thermal regulation through TH in phylogeny as well as ontogeny and propose a tradeoff between regeneration capabilities vs. high and regulated body temperature (Hirose et al., 2019). However, it seems that genetic determination of resource allocation to reproduction and differentiated tissue might be the main cause of loss of regeneration in mice, as poikilotherm anurans are also unable to regenerate the heart after metamorphosis, which is TH induced (Marshall et al., 2019). On the other hand, regenerative capabilities of another mammalian (thus homeotherm) species, *Acomys* (spiny mice), is much higher, speaking against a general rule of homeotherm-regeneration trade-off (Maden, 2018; Sandoval and Maden, 2020). Furthermore, it has been suggested that TH peaks coincide with becoming independent of parental care in mammals and sauropsids (Holzer and Laudet, 2013). Altricial (self autonomous at birth/hatching) species of birds and mammals show a peak of TH at birth/hatching. In contrast precocial (dependence on parental care at birth/hatching) species show a smoother increase in TH hormones during postembryonic development, which coincides with active thermoregulation, end of weaning and autonomous feeding (McNabb et al., 1984; Castro et al., 1986; Richardson et al., 2002; McNabb, 2006). Thus, TH peaks are associated with a “larva”-juvenile transition in homeotherm vertebrates and resembles the remnants of a metamorphosis element during postembryonic development.

Another important finding for TH signaling in homeotherm vertebrates was the control of seasonal gonadal development in Japanese quail to mediate optimally timed reproduction. The photoperiod is measured by TSH expression in the pars tubularis of the hypothalamus and controls the expression of deiodinases (D2 and D3) in the mediobasal hypothalamus, thereby increasing local T*3* concentrations about 10-fold. This local TH increase causes a morphological change in the axon terminals of GnRH producing neurons, which end there and increase their production and secretion of GnRH. This in turn increases the production of sex hormones and leads to temporal gonadal growth to orchestrate reproduction with the seasonal changes of light (Nakayama and Yoshimura, 2018). A similar axis of photoperiodic changes in local TH production was observed in melatonin proficient mice and suggests a similar regulation of seasonal developmental adjustments in mammals (Nakayama and Yoshimura, 2018). The effects of TH signaling due to different photoperiods can thereby be context specific, as for example two closely related vole species adapt seasonal strategies (growth vs. fast reproduction) in contrary direction, although both effects are regulated through the TH-signaling pathway (van Rosmalen et al., 2020).

### Evolution of Thyroid Receptors

Within vertebrates, the genes for the TRs diversified before the split of the gnathostome lineage and thus TRs and RXRs resemble the evolution of SR (see above) as their major diversification event is occurring at the genome duplication event during Teleost evolution (Escriva et al., 2002).

Cyclostomes are the evolutionary first branching animals within the vertebrate group. Although their TR and RXR repertoire is the same as in other vertebrates (two TR homologs and three RXR homologs), this seems to be due to convergent evolution. The sequences for both receptors cluster in monophyletic groups as outgroups to the rest of the vertebrate receptors (Escriva et al., 2002; Manzon et al., 2014). This might explain the difference in control of metamorphosis by TH (see above). However, it is interesting to see that the general signaling pattern evolved before the integration of developmental events in vertebrates, so that the TH signaling is important for metamorphosis, but the signal transduction can be implemented fundamentally differently (increase vs. decrease of TH). This hints to the importance of integrated cues within the TH-signaling, which are crucial for correct timing of postembryonal developmental events.

Outside vertebrates, there is clear evidence of TR signaling in Cephalochordata, which controls the metamorphosis in these animals through a single TR (Paris et al., 2008, 2010; Figure 2). The TR has the uncommon ligand Tiratricol (TRIAC), which is a derivative of T*3* (Paris et al., 2008) and is effectively deactivated by endogenous deiodinases (Klootwijk et al., 2011). The TRIAC synthesis requires an additional enzymatic step, indicating that TH-signaling evolved by reducing the biochemical processes on the synthesis of the hormone. Interestingly, the synthesis of active TH resembles again a breakdown process (deamination and decarboxylation, diodination in vertebrates), similar to the production of active steroid hormones. It seems that this is a general pattern for the evolution of NR ligands and makes sense in the light of NR evolution, as NRs turn from rather promiscuous receptors for a variety of compounds, which regulate metabolism and detoxification, to highly specific hormone receptors. It indicates that the active hormone evolves as a byproduct of already available biosynthetic pathways.

Within the other Deuterostomia, less is known about the evolution of TH-signaling. Tunicates and Echinoderm genomes contain a TR and form outgroups to the vertebrate clade, with echinoderm receptors showing more homology to vertebrate sequences (Ollikainen et al., 2006). This divergence is also reflected in the binding pocket for the ligand in these receptors, leading to the initial conclusion that classical TH are not able to bind in these animals (Ollikainen et al., 2006). Still, members of both clades are responsive to exogenous addition of TH in terms of acceleration of metamorphosis and development, show increase in TH levels before metamorphosis and expression of TRs (Taylor and Heyland, 2017). However, some of the echinoderm species seem to not produce TH by themselves (Chino et al., 1994), while others do (Heyland et al., 2004). The molecular actions of TH and the generality, that they are developmentally relevant in these species, is thus still unclear and needs further investigation (Figure 2).

Within the Protostomia, TRs have been identified in platyhelminths (Wu and LoVerde, 2019) and mollusks (Fukazawa et al., 2001; Huang et al., 2015; Wang et al., 2019; Li et al., 2020; Figure 2). Functional knockdown or knockout of the TR in the abalone *Haliotis diversicolor* and the oyster *Crassostrea gigas* reduced the proportion of metamorphic animals, indicating a role of thyroid signaling in postembryonal development in mollusks (Wang et al., 2019; Li et al., 2020). It was previously shown that after external administration of T*4*, metamorphosis could be induced in other species of abalone (Fukazawa et al., 2001) and that the oyster TR is responsive to TH treatment *in vivo*, although not *in vitro* (Fukazawa et al., 2001). However, the TR expression is peaking during the gastrulation of *C. gigas*, but shows only low expression during metamorphosis, speaking against a function in metamorphosis (Vogeler et al., 2016). Taken together, these results indicate a regulative function of TH in mollusk metamorphosis, although the effects are not as conclusive as in vertebrates.

Despite the findings in mollusks and the presence in platyhelminths, no TR could be identified in other Protostomia species. The presence of a TR in mollusks, however, implies a common ancestor in the pre-bilaterian lineage. This ancestral TR has been retained in Deuterostomia, Molluska, and Platyhelminthes but has probably been lost in the other Protostomia clades. In turn, this means that Annelida, Gnathifera, and Ecdysozoa have lost the TR independently, indicating a less vital role of TR in the urbilaterian ancestor of these animals (Figure 2). Given the fact that thyroid-like molecules are common in marine environments and that these are easily integrated into the food chain of the animals (Holzer et al., 2017; Markov et al., 2018), it is likely that the ancestral TR served as a nutrient sensor to control metabolism. During evolution, this sensory function might have become less important, which leads to either the loss of the signaling pathway, or the neofunctionalization to serve as a hormone-integrating metabolic and developmental function.

In Cnidaria, an RXR plays a major role in the transition from asexual reproduction of sessile polyps to sexual reproduction in pelagic medusae called strobilation of *Aurelia aurita* (Fuchs et al., 2014). In the same study, the authors identified a peptidergic ligand, which is the inducer of strobilation, while 9-cis-RA seems to be a strong coactivator (Fuchs et al., 2014). However, the dimerization partner for RXR is still elusive in this process and to date it is unknown how the peptide ligand is recognized in this strobilation event. However, the peptide inducing strobilation comprises WSRRRWL with the tryptophane residues being the inducing agent, as could be shown by chemical analogons (Fuchs et al., 2014). This is a striking similarity to the TH, which is derived from another aromatic amino acid—tyrosine. Hence, it might be possible that the putative cnidarian receptor and the TR have a common ancestor, which recognized peptidergic ligands.

## Ecdysone Receptor Signaling

### Evolution of Thyroid Receptors

Postembryonal development in Ecdysozoa is determined by several stages of larval development, which is accompanied with cuticle shedding to allow growth. Regulation of this growth period is necessary to attain the species specific body sizes and is associated with the onset of sexual maturity (Mirth and Shingleton, 2012). Thus, morphogenesis and sexual maturation coincides in insects and are not separate developmental steps, as in vertebrates, although they may be differentially regulated. In insects, this regulation is mediated by the production of ecdysone (20-hydroxecdysone and derivates) and juvenile hormone (JH) (Hiruma and Kaneko, 2013; Liu et al., 2018). The former is produced in pulses throughout the larval development and determines transitions between the different larval instars, while the latter is predominantly produced in the larval stages. If JH levels drop, ecdysone induces pupariation leading to fixation of final body size and sexual maturity (Mirth and Shingleton, 2012). While ecdysone is recognized by an NR, namely, the EcR (member of the LXR/NR1H group), JH is bound by methropen tolerant (met, a basic-helix-loop-helix PAS domain receptor/AhR homolog, which is functionally very similar to NRs) (Hiruma and Kaneko, 2013; Dubrovsky and Bernardo, 2014). The actions of both hormones are thus integrated at the genetic level. Ultraspiracle (USP, member of the RXR/NR2B group) serves as the coreceptor of EcR (Yao et al., 1992, 1993; Jones and Sharp, 1997).

The production of ecdysone is controlled by two main components: insulin-like peptides (ILPs) (Colombani, 2005) and PTTH (Shimell et al., 2018). Thereby, PTTH is responsive to ILP signals from imaginal disks to facilitate allometry and damage repair (Colombani et al., 2015; Garelli et al., 2015; Vallejo et al., 2015; Jaszczak et al., 2016). Additionally, ILP signals coordinate the integration of the nutritional state to PTTH and the ecdysone signaling (Faunes and Larraín, 2016). For pupariation, the larva needs to attain a certain weight (critical weight) to safely make the progression to the adult fly. The correct size at pupariation is monitored by the corpora allata, the production tissue of PTTH (Mirth et al., 2005; Shimell et al., 2018). Furthermore, PTTH integrates light signaling and photo periods to coordinate developmental timing to circadian rhythms (Mirth et al., 2005; Shimell et al., 2018) and forms a feed-forward loop with ecdysone signaling in the brain (Christensen et al., 2020). ILPs do not only act through the PTTH axis to mediate their effect on the ecdysone but are able to directly control the ecdysone production in the prothoracic gland (PG) (Colombani, 2005). ILPs signaling is regulated by extrinsic signals like temperature (Li and Gong, 2015) and nutrition (Hyun, 2013; Lee et al., 2018), which in turn influences the developmental timing in insects. PTTH and ILP are regulated by a complex network of neuronal signals mediated by neuropeptides and sense the mentioned extrinsic signals (Koyama et al., 2020). Interestingly, the signals of PTTH and ILP need priming of the PG by activin, a TGF-β member, before they are able to induce ecdysone production (Gibbens et al., 2011). Furthermore, other environmental factors like oxygen are integrated in the ecdysone signaling (Callier and Nijhout, 2011), rendering it a highly environmentally dependent decision point for postembryonal development adjusting life history traits to the given environment.

Remarkably, the EcR itself induces expression of E75 (Rev-ERB/NR1 subfamily), E78 (Rev-ERB/NR1 subfamily), DHR3 (ROR/NR1 subfamily), FTZ-F1 (SF-1/NR5 subfamily), DHR39 (SF-1/NR5 subfamily), and DHR4 (GCNF/NR6 subfamily) upon activation by ecdysone—all belonging to the NR class of transcription factors. These factors control and execute correct molting and pupariation, next to other essential functions during embryonic development (Richards, 1997).

*Caenorhabditis elegans* possesses an alternative steroid signaling pathway that involves dafachronic acid (DA) and its receptor dauer formation 12 (DAF12, LXR/NR1 subfamily member) (Fielenbach and Antebi, 2008), a closely related NR to EcR. DAF12 regulates the occurrence of an additional senescent larval state, dauer diapause, and thereby developmental timing, reproductive maturation, metabolism, and lifespan. Dauer formation is initiated upon detrimental environmental conditions, such as starvation, high temperature, or high aggregation of worms (Fielenbach and Antebi, 2008), which prevents the synthesis of DA (Motola et al., 2006). Many (if not all) of the environmental cues are transduced by two main signaling cascades—the insulin and the TGF-β pathway—which form the most important regulators for DA in *C. elegans* (Fielenbach and Antebi, 2008). Dauer forms are resistant to all kinds of environmental stresses and can extend lifespan up to 3–6 months. Once the environmental conditions become more favorable, *C. elegans* resumes its development to sexual maturation and reproduction (Fielenbach and Antebi, 2008). The regulation, the mode of action, and the signaling outcome is thus very similar to the ecdysone system in insects and might resemble an evolutionary special case of *C. elegans*, where DA replaced the generic ecdysone (Gáliková et al., 2011) thereby regulating genes homologous to the EcR downstream genes (Gissendanner et al., 2004).

### Evolution of Ecdysone Receptors

The ecdysone signaling pathway is one of the best understood hormonal pathways in invertebrate species, not least because of *Drosophila melanogaster*’s role as a pivotal model organism (Koelle et al., 1991). Within the Ecdysozoa, the number of isoforms differ in the different clades. While *Locusta* inherits only one isoform, *Drosophila* expresses three isoforms with different function, although expressed from a single locus (Truman and Riddiford, 2002).

Ecdysone receptor is uniformly found in most ecdysozoan species, except to the nematode *C. elegans* (Schumann et al., 2018), which lacks EcR and USP genes. However, the NRs, which are downstream of the EcR in insects and that are usually involved in molting, exist and have similar functions in *C. elegans* as well (Gissendanner et al., 2004). Hence, the loss of ecdysone and its receptor might be very specific to *C. elegans.*

Interestingly, DAF12, the DA receptor of *C. elegans* is similarly closely related to the EcR, but seems to have been evolved only in a rather *C. elegans* specific clade (Sluder, 2001). At least filarial nematodes possess a functional ecdysone signaling, indicating the EcR ancestry in nematodes (Tzertzinis et al., 2010; Mhashilkar et al., 2016). However, the definitive evolutionary trajectory of EcR in the nematode lineage remains to be clarified.

While the expression of EcR seems to be an ancestral state of Ecdysozoa, the enzymes for the synthesis of ecdysone (so-called Halloween genes) are not found in all subclades and species. There is a stepwise evolution of ecdysone producing CYP450 enzymes: Nematoda and Priapulida do not contain any of these genes, while Tardigrada and Onychophora express a *sad* gene. In the Panarthropoda, the genes *spook*, *disembodied*, and *shadow* are additionally found. From there, the additional expression of *phantom*, *spookiest*, and *spok* are found in a stepwise addition in Myriapoda (centipedes), Crustacea, and Hexapoda (Schumann et al., 2018). It is thus interesting to see whether and how other ecdysone derivatives are produced in other animal clades within the Ecdysozoa. It might elucidate the evolution of Ecdysone signaling in the Ecdysozoa and by that will provide valuable information to the evolution of a steroid ligand in all animals.

It has long been thought that ecdysone signaling was a first insect, later ecdysozoan invention only (Sluder, 2001; Hyde et al., 2019), but newer studies found EcRs outside the Ecdysozoa. Annelida, Molluska (Lophotrochozoa), and Platyhelminthes contain an EcR homolog in their genome, although not much is known of the function of this receptor in these species (Laguerre and Veenstra, 2010; Vogeler et al., 2014; Kaur et al., 2015). There is an upregulation of EcR to the onset of metamorphosis in the mollusk *C. gigas* (Vogeler et al., 2016) but apart from that, functional studies are lacking.

The EcR is closely related to the liver X receptor (LXR) in humans and even more closely to the one found in *Ciona intestinalis*, which indicates a common ancestry of the two receptors (Truman and Riddiford, 2002; Figure 2). Both receptors are known to bind steroid ligands, while the deuterostome LXR is involved in metabolic regulation for cholesterol and binds oxysterol (Lehmann et al., 1997; Yoshikawa et al., 2001), EcR evolved into a major determinant of postembryonal development in arthropods binding ecdysone. The common ancestry, however, indicates a function of steroid-binding NRs before the split of Deuterostomia and Protostomia. It would be highly interesting to investigate the nature and the regulatory function of such a receptor to elucidate the evolution of steroid signaling. Was the hormone function of steroids acquired in insects or lost in deuterostomes?

## Retinoid X Receptors—Important Coreceptor of NRs in Postembryonal Development

Retinoid X receptors have a special role during the signaling of different NRs, because they form the heterodimerization partner for many of the NRs and hence enable their signaling. RXR-heterodimers are formed with TR, VDR, RAR, PPAR, LXR, farnesyl X receptor (FXR), pregnane X receptor (PXR), or constitutive androstane receptor (CAR)—all NR1 subfamily members (Desvergne, 2007; Evans and Mangelsdorf, 2014). RXR are thereby able to bind a variety of endogenous and natural occurring compounds, including 9-cis retinoic acid (9-cis RA), linoleic acid (and other unsaturated fatty acids), and phytanic acid (Dominguez et al., 2017). These compounds are all readily available as nutrients and RXR therefore is an important hub for the integration of nutritional information into metabolic and developmental pathways. However, despite the name of the receptor, there is considerable doubt on the physiological relevance of 9-cis RA as ligand for RXR (Mic et al., 2003; Calleja, 2006; Dawson and Xia, 2012) and it seems more probable that 9-cis-13,14-dihydroretinoic acid is an endogenous ligand (Rühl et al., 2015), while various fatty acid ligands obtained through the diet might be relevant RXR ligands (Dominguez et al., 2017).

Depending on the interaction partner, liganded RXR either activate signaling or enhance it. The dimerization partners can be classified in permissive and nonpermissive. Hence, ligand binding in either of the dimers is sufficient to activate signaling (permissive), or signaling is activated only if the dominant partner (not RXR) is liganded (nonpermissive) (Germain et al., 2002; Desvergne, 2007). Interestingly, this classification coincides with the specificity and binding affinity strength of the ligand to the receptor. Nonpermissive NRs are highly specific for their ligand, hence exhibit a strong binding affinity. Nonpermissive receptors include TR, VDR, and RAR. They fulfill crucial developmental signaling and recognize endogenous hormones. Additional binding of a ligand to the RXR generally enhances the signaling strength, thus forms an option for modulating the signaling outcome (Germain et al., 2002).

Nonpermissive signaling seems crucial already in evolutionary early branching organisms like Cnidaria, where it promotes strobilation in *A. aurita* (Fuchs et al., 2014). In *T. adhaerens*, the supplementation of 9-cis RA in the food modulates growth and shape, which is recognized by the RXR (Novotný et al., 2017). The fly RXR homolog *usp* is the binding partner of the EcR (Yao et al., 1992; Yao et al., 1993). Although 9-cis RA seems not to be a relevant ligand for *usp* (Oro et al., 1990), correct formation of the ligand binding pocket in *usp* is necessary for normal larva to adult transition in *Drosophila*, thus Usp mediates another control point for correct development (Jones et al., 2013). In vertebrates, TR-RXR dimer mediate morphogenesis and specific inhibitors/activators for RXR are able to abrogate/enhance precocious metamorphosis under T*3* treatment (Mengeling et al., 2018). Given the range of RXR ligands and its modulating role in metamorphosis, it is very likely that RXR has a function in coordination of major developmental steps to the nutritional state of the organism. However, the current data shows only insufficiently if and how RXR relay this information to developmental decisions.

Peroxisome proliferator-activated receptor, LXR, FXR, and PXR are permissive NRs and exhibit a broader range of possible ligands with a much lower binding affinity for them. However, they play important roles in detoxification and regulation of metabolism (Desvergne, 2007; Duniec-Dmuchowski et al., 2007; Lim and Huang, 2008).

## The Role of CYP Enzymes in NR Regulation and Function

Cytochrome P450 proteins are a class of oxidizing enzymes, which have a broad range of substrates. They play a pivotal role in metabolizing hydrophobic molecules by oxygenation and thus render them more hydrophilic for subsequent function in metabolism and signaling (Danielson, 2002).

CYP enzyme activity is closely regulated in the interplay with NRs. On the one hand, most of the ligands of the NRs are synthesized by at least one CYP enzyme (Miller, 1988; Cheng et al., 2004; Motola et al., 2006), while on the other hand many, if not all NRs regulate the expression of CYP enzymes after activation (Honkakoski and Negishi, 2000). This tight interaction forms feedback mechanisms within the regulation of NRs and renders CYP enzymes extremely important for environmental signal integration. They form key regulatory steps for the production or catabolism of hormones, which control developmental decision points (Miller, 1988; Cheng et al., 2004; Gilbert, 2004; Motola et al., 2006; Catharine Ross and Zolfaghari, 2011).

Although tightly integrated in the NR network, CYP enzymes appeared earlier in evolution than NRs and are present in all kingdoms of life (Danielson, 2002; Nelson, 2018) in contrast to NRs, which can be found only in metazoans (Bridgham et al., 2010). The ancestral function for CYPs was not necessarily associated with metabolism of xenobiotics but rather part of the physiological metabolism (Bridgham et al., 2010). In extant species, most of the CYP enzymes are involved in either metabolization of xenobiotics or the production of structural or signal molecules (Sezutsu et al., 2013) and can be functionally distinguished into environmental response genes or physiological metabolic regulators (Sezutsu et al., 2013).

In metazoans, CYP enzymes can be roughly classified into 10–11 major classes, so-called clans: CYP-clan 2, 3, 4, 7, 19, 20, 26, 46, 51, 74, and mito (-chondrial) (Gotoh, 2012; Nelson et al., 2013). These have evolved mainly by gene duplication events, which led to blooming of some of the clans with many similar enzymes and a broad range of substrates (Nelson et al., 2013). However, clans that contain genes, which are associated with hormone synthesis are usually small, with only one or a few members, indicating more evolutionary constraints for these genes (Thomas, 2007). Furthermore, these clans are generally rather derived and appeared late in evolution, like the enzymes for the steroid synthesis, which can only be found in phyla leading to vertebrates (Gotoh, 2012) or ecdysone-producing enzymes in Arthropoda (Markov et al., 2009).

In general, CYP enzymes belong to the fastest evolving genes and there is not a single residue conserved across this group of genes (Danielson, 2002; Sezutsu et al., 2013) and even the number of members in the different clans, as well as the number of clans present in the different phyla is highly variable (Nelson et al., 2013). Even developmental important genes like CYP307, which is involved in ecdysone production of arthropods, are highly unstable. Several paralogues of CYP307 were independently lost and gained within the arthropod clade (Sezutsu et al., 2013) and it exemplifies that the genetic plasticity can cause the adaption of developmental processes to the environment on the genetic level.

## From Environmental Sensor to Developmental Determinant

It is worth mentioning that it is no coincidence that the NRs form these conserved signaling molecules, which regulate postembryonal developmental transitions. A main evolutionary argument for distinct life cycles in organisms has been the separation of ecological niches in larval and adult forms (Holstein and Laudet, 2014). The timing of the transitions between these two states crucially depends on two factors: the developmental state of the larva and the environmental conditions. Is the larva not developed well enough (too small in most of the cases), it will not survive the transition, because energy reserves are not sufficient to facilitate the tissue remodeling during metamorphosis (De Moed et al., 1999; Laudet, 2011; Gokhale and Shingleton, 2015). On the other hand, if environmental conditions are unfavorable for the transition, the animal might mature in an environment inappropriate for sexual reproduction. This integration of environmental cues into developmental pathways has been termed phenotypic plasticity and was determined to be a major driver of evolution (West-Eberhard, 2003; Gilbert and Epel, 2015).

**FIGURE 4:**
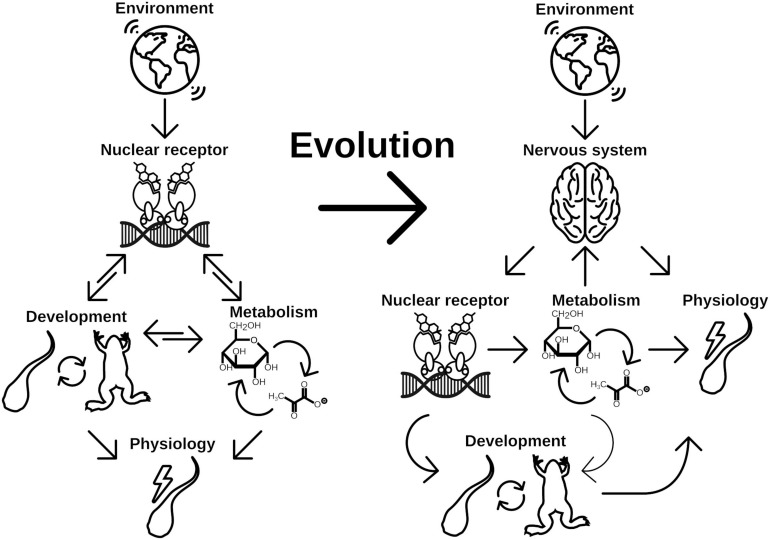
Evolution of NRs as central point for developmental switch control is a consequence of early implementation of physiology regulation. Physiological response to different environmental cues were initially controlled by two main regulation routes, metabolism and life history changes. While changes in metabolism is the immediate response to given conditions, developmental switches control the time point of ecological niche changes. Both routes might be directly controlled by NRs in early branching animals, which renders the NRs a central element for phenotypic plasticity. With evolution of the nervous system, the evolutionary constrains on the NRs for direct control of physiology has been lifted. Consequently evolution of hormone signaling was enabled, which controls developmental processes independent of direct environmental inputs. Environmental cues are recognized by the nervous system, which eventually controls hormone signaling (thus NR signaling) and at the same time is able to directly control aspects of physiology of the organism. It thus adds another layer of environmental signal processing to facilitate more fine grained and at the same time more flexible control of physiology and life history decisions, which increases the phenotypic plasticity of the organism.

The first checkpoint is generally closely regulated by endogenous control of the metabolism and growth factors like mTOR or insulin signaling (Gokhale and Shingleton, 2015). The second factor—environment—is less well defined and is highly specific to the respective organism and can range from various cues like population densities (Golden and Riddle, 1982; Zwaal et al., 1997), temperature (Leatherland et al., 1990; Kingsolver and Huey, 2008; Fuchs et al., 2014; Politis et al., 2018), photoperiod (Nakayama and Yoshimura, 2018), or bacterial status (Hadfield, 2011). Integration of these diverse signals necessitates the evolutionary flexibility of NRs and the associated CYP enzymes both in terms of ligand and substrate recognition, respectively. This is especially true for organisms without a functional nervous system (basically Placozoa and Porifera) as there is no special tissue dedicated to the recognition for extrinsic signals. During evolution, the flexibility was early integrated, first into the control of metabolism (Bridgham et al., 2010) and later into developmental pathways (Novotný et al., 2017; Figure 4). Once integrated into the developmental pathways, the NRs were evolutionary fixed and thus relatively stable in their function to form the transitional switch. However, with the evolution of the nervous system as an even more flexible system for environmental integration emerged, allowing direct physiological responses (Arendt et al., 2016). However, this freed the original ligand of the NR from evolutionary restrictions and enabled the organism to neofunctionalize the ligand-binding properties (Markov and Laudet, 2011; Figure 4). The neuronal signals were again integrated in the production of NR ligands controlling the developmental switches, which is reflected in the HPT of vertebrates and the corpora allata—pituitary gland axis in insects, which are strikingly similar (Figures 3, 4).

Although this argumentation is sound in itself, experimental evidence lacks for such a scenario. It would be interesting to further investigate the mechanisms of life history changes in early emerging animals, such as Porifera, Placozoa, and Cnidaria (Bosch et al., 2014). Hereby, Cnidaria take an extraordinary role, as they developed a rudimentary nerve cell system, which forms the prototype for nervous system functions for all other animals (Klimovich and Bosch, 2018). There are intricate interactions between the nervous system of *Hydra* and its associated microbiome (Augustin et al., 2017; Murillo-Rincon et al., 2017). and we have shown that the microbiome controls developmental programs *via* Wnt (Taubenheim et al., 2020). It thus seems that the cnidarian nervous system is able to integrate environmental signals, like the associated bacteria, into developmental pathways. Similarly, Wnt and TGF-β include temperature and metabolic information *via* insulin signaling to control body size in *Hydra* due to timing of asexual reproduction (Mortzfeld et al., 2019). That again links back to resource allocation between reproduction and growth. It is highly analogous to the control of maturation in insects *via* ecdysone or in vertebrates *via* estrogen. Hence, it would not be surprising to see an NR controlling the switch between growth and reproduction in Cnidaria, which would elucidate the evolutionary trajectories of NR signaling in pre-Bilateria. Given the fact that *A. aurita* controls sexual maturation (strobilation) *via* an RXR associated process (Fuchs et al., 2014), it is indeed very likely.

Furthermore, elucidation of ligands for NRs in early emerging metazoa would be interesting, because it would shape our notion on how hormonal ligands and their synthesis pathway evolve. It seems less surprising that the synthesis pathways for the functional hormonal ligands resemble catabolic processes like in estrogen production (Payne and Hales, 2004), activation of TH (deiodination) or the ecdysone production, a multiple oxidated cholesterol derivative and typical for detoxification of xenobiotics (Liska, 1998). Steroid derivatives emerged at least twice independently during evolution (Markov et al., 2009), probably because of diverse possibilities to modify the steroid backbone and its conformation (Brueggemeier and Li, 2010).

Taken together, it is rather a consequence of evolutionary constraints, than coincidence that NRs are central to major postembryonal developmental processes. However, ancestral functionality, the integration of the diverse environmental, as well as intrinsic cues into these pathways, may it be due to sensory neurons or the signaling by growth or metabolic factors, is insufficiently understood across the animal tree of life. To study these systems in non-model organisms, especially on the brink of evolution of nerve systems and bilaterality, promises insights in the evolution of different life histories. In turn, this promises nothing less than to understand a major driver of ecological adaptation, animal diversity, and the mechanisms of speciation.

## Author Contributions

JT and CK reviewed the literature. JT, CK, and SF wrote the manuscript. JT and SF supervised and conceptualized the project. All authors contributed to the article and approved the submitted version.

## Conflict of Interest

The authors declare that the research was conducted in the absence of any commercial or financial relationships that could be construed as a potential conflict of interest.
